# Prognostic Value of the Sum of Metabolic Tumor Volume of Primary Tumor and Lymph Nodes Using ^18^F-FDG PET/CT in Patients With Cervical Cancer

**DOI:** 10.1097/MD.0000000000002992

**Published:** 2016-03-07

**Authors:** Jin Hwa Hong, Kyung Jin Min, Jae Kwan Lee, Kyeong A So, Un Suk Jung, Sungeun Kim, Jae Seon Eo

**Affiliations:** From the Department of Obstetrics and Gynecology, Guro Hospital, College of Medicine, Korea University (JHH, KJM, JKL); Department of Obstetrics and Gynecology, Cheil General Hospital and Women Healthcare Center, Dankook University College of Medicine (KAS); Department of Obstetrics and Gynecology, Hallym University Hangang Sacred Heart Hospital (USJ); Department of Nuclear Medicine, Anam Hospital, College of Medicine, Korea University (SK) and Department of Nuclear Medicine, Guro Hospital, College of Medicine, Korea University, Seoul, Korea (JSE).

## Abstract

This is an observational study to determine the most relevant parameter of ^18^F-fluorodeoxyglucose (FDG) positron emission tomography/computed tomography (PET/CT) for predicting recurrence in cervical cancer.

Fifty-six patients with International Federation of Gynecology and Obstetrics (FIGO) stage IIB-IVA cervical cancer who underwent pretreatment ^18^F-FDG PET/CT were enrolled. PET parameters including maximum standardized uptake value (SUVmax), metabolic tumor volume (MTV), and total lesion glycolysis (TLG) of both primary tumor and pelvic and/or para-aortic lymph nodes were analyzed. SUVmax-S was defined as the sum of the SUVmax of primary tumor and the higher SUVmax of either pelvic or para-aortic lymph nodes. MTV-S was defined as the sum of the MTV of primary tumor and pelvic and para-aortic lymph nodes. TLG-S was calculated in the same way as MTV-S. We evaluated the relationship between these PET parameters and recurrence-free survival (RFS).

Univariate analysis revealed that higher FIGO stage (hazard ratio [HR] = 5.61, 95% confidence interval [CI]: 1.68–18.68, *P* = 0.005), lymph node metastasis (HR = 3.42, 95% CI: 1.08–10.84, *P* = 0.037), MTV of primary tumor >47.81 cm^3^ (HR = 6.20, 95% CI: 1.35–28.48, *P* = 0.019), TLG of primary tumor >215.02 (HR = 11.82, 95% CI: 1.52–91.96, *P* = 0.018), MTV-S > 59.01 cm^3^ (HR = 8.24, 95% CI: 1.80–37.77, *P* = 0.007), and TLG-S > 224.15 (HR =  13.09, 95% CI: 1.68–101.89, *P* = 0.014) were associated with RFS. In multivariate analysis, FIGO stage (HR = 4.87, 95% CI: 1.38–17.18, *P* = 0.014) and MTV-S > 59.01 cm^3^ (HR = 7.37, 95% CI: 1.54–35.16, *P* = 0.012) were determined to be independent predictive factors for RFS.

Our preliminary results reveal that MTV-S is an independent prognostic factor for RFS in patients with cervical cancer treated by definitive chemoradiotherapy.

## INTRODUCTION

Cervical cancer is the third most frequently diagnosed gynecologic cancer and the fourth leading cause of cancer death worldwide.^1,2^ Cervical cancer is clinically staged according to the International Federation of Gynecology and Obstetrics (FIGO) classification system. Although imaging does not feature within this system, it is frequently used to determine tumor size, parametrial involvement, lymph node metastasis, or distant metastasis. As most patients with locally advanced cervical cancer are treated with definitive chemoradiotherapy, identifying the disease extent, especially lymph node involvement, is crucial. Patients with FIGO stage IB-IIA have a recurrence rate ranging from 10% to 20%, whereas patients with FIGO stage IIB-IV have a 50% to 70% chance of recurrence.^3^ Several clinical and pathological risk factors that are predictive of disease outcome have been identified, including age, FIGO stage, tumor histology, tumor size, and pelvic and/or para-aortic lymph node metastasis.^4–6^ Recently, ^18^F-fluorodeoxyglucose (FDG) positron emission tomography/computed tomography (PET/CT) has played an increasing role in staging and monitoring gynecologic cancers. The clinical value of ^18^F-FDG PET/CT has been evaluated and confirmed in terms of pretreatment staging, tumor localization, and surveillance of post-treatment response in previous literature.^7–9^ In addition to localizing tumor extent, ^18^F-FDG PET/CT gives useful information regarding disease prognosis through its semiquantitative parameter, such as standardized uptake value (SUV), and volumetric parameters, such as metabolic tumor volume (MTV) and total lesion glycolysis (TLG).^10–12^ Although the prognostic value of ^18^F-FDG PET/CT in cervical cancer has been demonstrated, some limitations still exist, for example, the relatively small number of studies, the major focus on the primary tumor, and conflicting results.^13–16^ Furthermore, there is still controversy in the literature regarding which metabolic parameters have a higher prognostic potential.^17–20^

The objective of this study was to determine the most valuable parameter of ^18^F-FDG PET/CT for predicting recurrence in locally advanced cervical cancer. For this purpose, we created novel parameters representing the metabolic activities of both primary tumor and metastatic pelvic and/or para-aortic lymph nodes. The role of these novel parameters, as well as maximum SUV (SUVmax), MTV, and TLG of primary tumor, in predicting recurrence was investigated.

## MATERIALS AND METHODS

The study protocol was approved as retrospective observational study by the Institutional Review Board for Research on Human Subjects at Guro Hospital, Korea University (KUGH15043-001). From August 2007 through April 2014, 56 patients with stage IIB-IVA cervical cancer who underwent ^18^F-FDG PET/CT scan were enrolled and analyzed. The patients were staged according to the FIGO staging system. All patients underwent routine clinical staging, including physical and gynecologic examination, complete blood count, blood chemistry tests, and CT or MRI of the abdomen and pelvis. The inclusion criteria were as follows: histologically confirmed, locally advanced primary cervical cancer (FIGO stage IIB-IVA); definitive chemoradiotherapy as a primary treatment modality; ^18^F-FDG PET/CT scan performed before primary treatment. The exclusion criteria were as follows: previous or concurrent diagnosis of any other primary malignancy; both previous and current inflammatory bowel disease or intra-abdominal infection; patients not assessed by ^18^F-FDG PET/CT before treatment; diagnosis of diabetes mellitus; follow-up duration ≤3 months.

### PET/CT Technique

All patients were imaged using a dedicated PET/CT system (Gemini TF 16; Philips Medical Systems, Cleveland, OH). Patients were instructed to fast for at least 6 hours before intravenous administration of 5 to 6 MBq/kg FDG. Blood glucose level was measured before administration of the radiotracer to ensure that it was <150 mg/dL. During the distribution phase, patients were kept lying comfortably in a quiet room. During uptake time, the patients were orally hydrated (500 mL of water) and were asked to empty their bladder immediately before the scan to reduce urinary bladder activity. Combined image acquisition began 1 hour after FDG administration. Unenhanced CT (4-mm slice thickness) was performed before PET from the skull base to the mid-thigh using a standardized protocol (140 kV and 80 mA). The subsequent PET scan was performed with a 1-minute emission acquisition per bed position. The images were acquired from the skull base to the mid-thigh without repositioning the patient. Attenuation was corrected using the CT images. PET images were reconstructed iteratively by applying the CT images for attenuation correction, and coregistered images were displayed on a workstation.

### Image Analysis

PET image analysis was performed on a dedicated workstation (Extended Brilliance Workspace 4.0, Philips Medical Systems, Cleveland, OH). Two board-certified nuclear medicine physicians analyzed the images. Discrepancies between the readers were resolved by a consensus. FDG uptake in both the primary tumor and the lymph nodes was quantitatively assessed using SUV normalized to body weight as a measure of tumor glucose metabolism. The SUVmax for primary tumor and pelvic and/or para-aortic lymph nodes were calculated. The contour around the target lesions inside the boundaries was automatically produced and voxels presenting SUV intensity ≥2.5 within the contouring margin were incorporated to define the MTV.^21^ TLG was defined as the MTV multiplied by the SUVmean. MTV and TLG of lymph nodes were calculated as the sum for all pelvic and para-aortic lymph nodes. SUVmax, MTV, and TLG of primary tumor were recorded as SUVmax-T, MTV-T, and TLG-T. SUVmax-S was defined as the sum of the SUVmax of primary tumor and the higher SUVmax of either pelvic or para-aortic lymph nodes. MTV-S was defined as the sum of the MTV of primary tumor and pelvic and para-aortic lymph nodes. TLG-S was calculated in the same way as MTV-S.

### Treatment

Patients were treated with a combination of external beam radiation with concurrent either weekly cisplatin 30 mg/m^2^ or triweekly paclitaxel 135 mg/m^2^ and carboplatin area under the curve (AUC) 5 and then high-dose-rate brachytherapy. A total of 54 Gy external radiation (daily 2 Gy per fraction) was delivered. Radiation field was extended in patients with FDG uptake in para-aortic lymph nodes. High-dose-rate brachytherapy was performed using 3.5 Gy per fraction, given in 8 fractions.

### Follow-up

Patients were followed every 3 months for 2 years, then every 6 months up to 5 years, and annually thereafter. At each visit, physical examination, imaging study, and serum tumor marker were taken. Recurrence was identified through biopsy or imaging studies.

### Statistical Analysis

All statistical analyses were performed using SPSS software (Version 19.0; SPSS Inc., Chicago, IL). The primary endpoint was recurrence-free survival (RFS) and the secondary endpoint was overall survival (OS). The RFS was calculated from the date of initiation of treatment to the date of recurrence, censoring, or last follow-up examination. OS was calculated from the date of initiation of treatment to the date of death, censoring, or last follow-up examination. Survival curves were generated using the Kaplan-Meier method and were compared with log-rank test. With respect to SUVmax, MTV, and TLG, receiver-operating characteristic (ROC) curve analysis was performed to determine the cutoff values for predicting recurrence. The optimal cutoff values of SUVmax, MTV, and TLG were those giving the highest sum of sensitivity and specificity. Univariate and multivariate analyses of clinicopathological factors and PET parameters were performed using the Cox proportional hazards regression model. Correlations among the PET parameters were analyzed using Spearman Rank correlation analysis. *P* values <0.05 were considered to be statistically significant.

## RESULTS

A total of 56 patients were enrolled in this study. Patient characteristics are listed in Table 1. The majority of enrolled patients had FIGO stage IIB, and most had squamous cell carcinoma. All patients received definitive chemoradiotherapy. Forty (71.4%) patients completed 3 cycles of chemotherapy and the remaining 16 (28.6%) completed at least 4 cycles of chemotherapy. More than 50% of the patients had pelvic lymph node metastasis identified on pretreatment imaging. All 12 patients with para-aortic lymph node metastasis had concomitant pelvic lymph node metastasis.

**TABLE 1:**
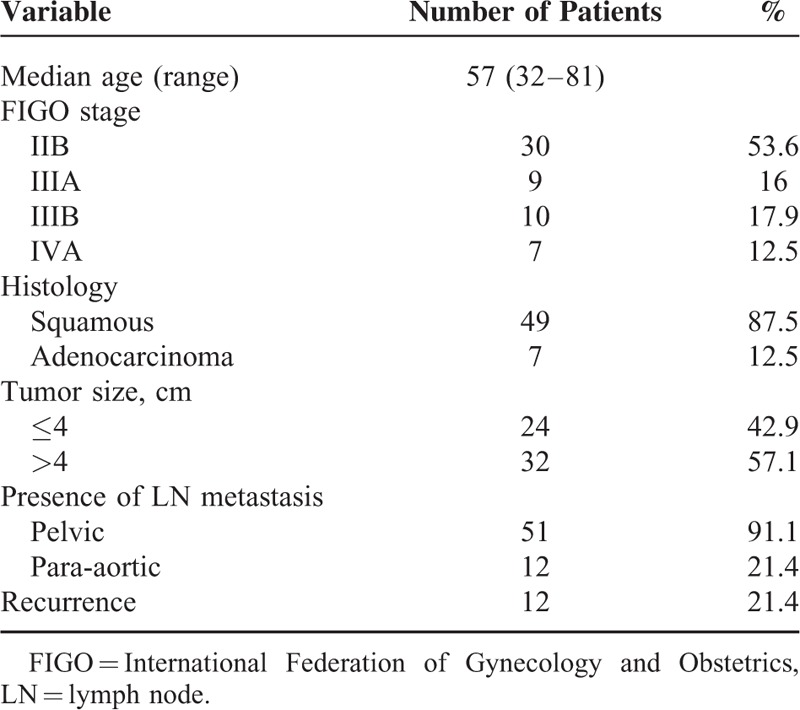
Baseline Characteristics of Enrolled Patients

The median follow-up was 20 months (range, 6–63 months). During the study period, recurrence occurred in 12 patients. Of these, 6 (50%) experienced pelvic recurrence, 5 (42%) had distant recurrence, and 1 (8%) had both pelvic and distant recurrence. The locations of distant recurrences were lung, supraclavicular lymph nodes, and para-aortic lymph nodes. At the time of the last follow-up, 36 (64.3%) were alive without disease, 16 (28.6%) were alive with disease, and 4 (7.1%) had died.

Given that RFS was the primary endpoint, the cutoff values of SUVmax, MTV, and TLG that showed the best trade-off between sensitivity and specificity for RFS were determined by ROC curve analysis. The AUC of SUVmax-T, MTV-T, TLG-T, SUVmax-S, MTV-S, and TLG-S were 0.463 (*P* = 0.697; 95% confidence interval [CI]: 0.30–0.63), 0.718 (*P* = 0.022; 95% CI: 0.55–0.88), 0.697 (*P* = 0.038; 95% CI: 0.53–0.86), 0.563 (*P* = 0.509; 95% CI: 0.42–0.70), 0.716 (*P* = 0.017; 95% CI: 0.58–0.83), and 0.699 (*P* = 0.031; 95% CI: 0.56–0.81), respectively. The best cutoff values of SUVmax-T, MTV-T, TLG-T, SUVmax-S, MTV-S, and TLG-S were 7.95, 47.81 cm^3^, 215.02, 9.60, 59.01 cm^3^, and 224.15, respectively.

**FIGURE 1:**
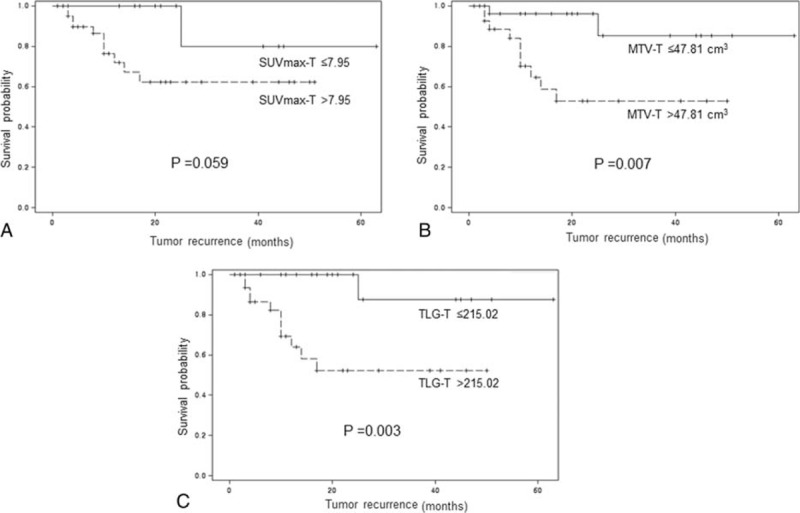
Kaplan-Meier curves for recurrence-free survival according to the cutoff values of SUVmax-T (A), MTV-T (B), and TLG-T (C).

**FIGURE 2:**
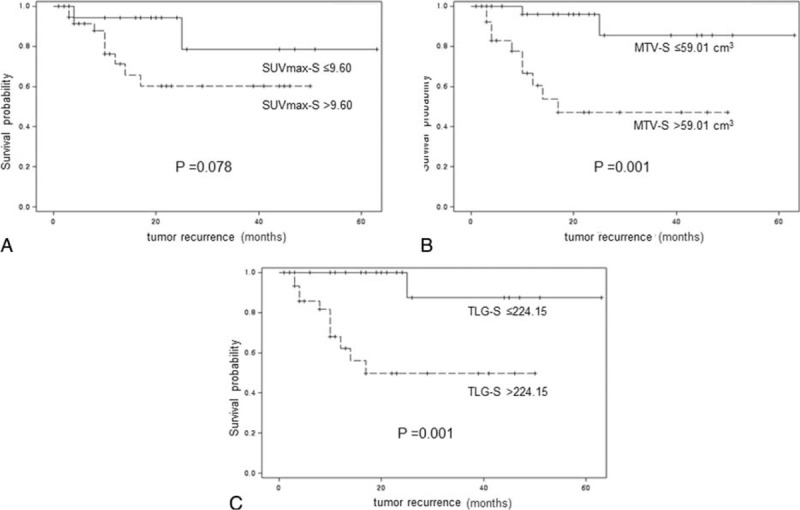
Kaplan-Meier curves for recurrence-free survival according to the cutoff values of SUVmax-S (A), MTV-S (B), and TLG-S (C).

Univariate analyses of prognostic factors for RFS are summarized in Table 2. Higher FIGO stage, presence of lymph node metastasis, MTV-T >47.81 cm^3^, TLG-T > 215.02, MTV-S > 59.01 cm^3^, and TLG-S > 224.15 showed a significant association with poorer RFS (*P* = 0.005, *P* = 0.037, *P* = 0.019, *P* = 0.018, *P* = 0.007, and *P* = 0.014, respectively). In multivariate analyses, FIGO stage and MTV-S > 59.01 cm^3^ were independent prognostic factors of RFS (*P* = 0.014 and *P* = 0.012, respectively) (Table 3). In terms of OS, no statistically significant factor was identified by univariate and multivariate analysis. The Kaplan-Meier curves for RFS are shown in Figures 1 and 2. Patients who met any of the following criteria of MTV-T ≤ 47.81 cm^3^, TLG-T ≤ 215.02, MTV-S ≤ 59.01 cm^3^, or TLG-S ≤ 224.15 showed better RFS (*P* = 0.007, *P* = 0.003, *P* = 0.001, and *P* = 0.001, respectively). Patients with SUVmax-T ≤ 7.95 or SUVmax-S ≤ 9.60 showed a trend of better RFS, although it was not statistically significant (*P* = 0.059 and *P* = 0.078, respectively).

**TABLE 2:**
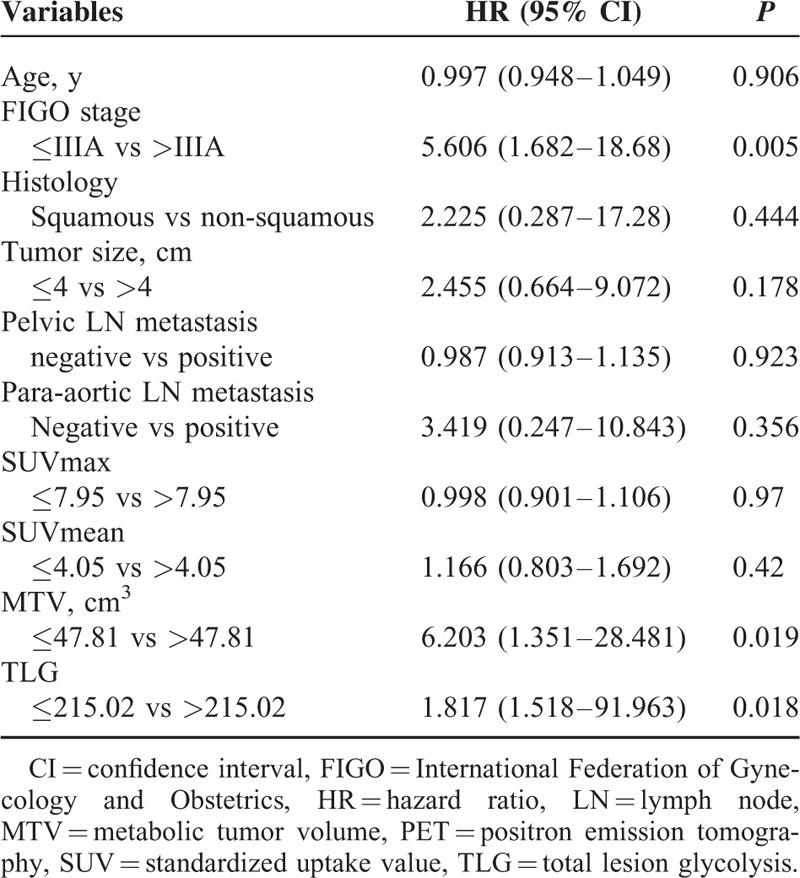
Univariate Analyses of PET Parameters and Clinical Factors for Recurrence-free Survival

**TABLE 3:**
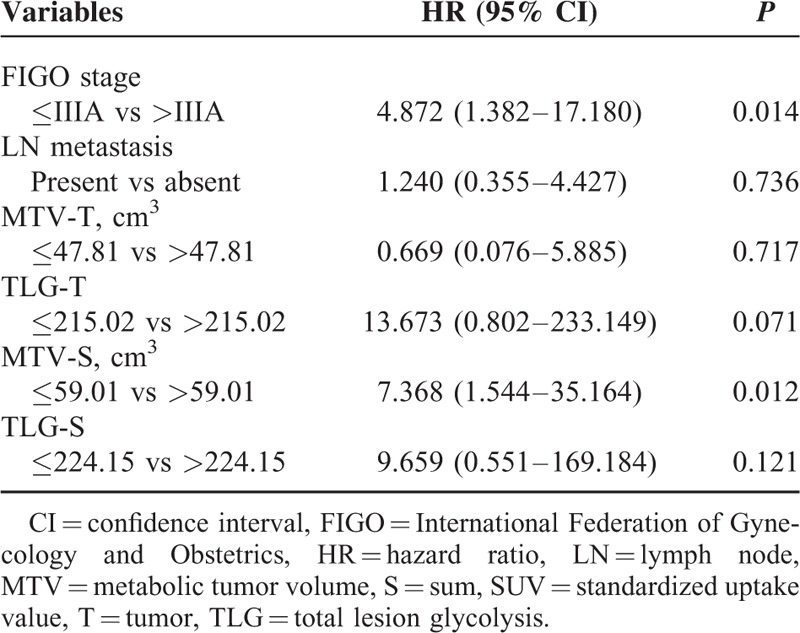
Multivariate Analysis of PET Parameters and Clinical Factors for Recurrence-free Survival

MTV-S showed a strong correlation with SUVmax-S, TLG-S, MTV-T, and TLG-T (Spearman Rank coefficient: 0.617, 0.979, 0.983, and 0.968, respectively; *P* = 0.000 for all parameters). There was a moderate correlation between MTV-S and SUVmax-T (Spearman rank coefficient = 0.432; *P* = 0.001).

## DISCUSSION

The present study investigated the prognostic role of various PET parameters in patients with locally advanced cervical cancer who underwent definitive chemoradiotherapy. Specifically, we aimed to identify the most valuable parameters for predicting recurrence using both traditional PET parameters (SUVmax-T, MTV-T, and TLG-T) and novel parameters (SUVmax-S, MTV-S, and TLG-S). To the best of our knowledge, this is the first study to examine the prognostic significance of SUVmax-S, MTV-S, and TLG-S in patients with locally advanced cervical cancer. Previous studies investigating the feasibility of ^18^F-FDG PET/CT for identifying recurrence and predicting disease outcome in patients with cervical cancer have mainly focused on the metabolic activity of the primary tumor. Chung et al^16^ analyzed 75 patients with FIGO stage IB to IIA cervical cancer and found that those with high SUVmax of the primary tumor showed an increased rate of recurrence. A higher pretreatment SUVmax or MTV of primary tumor has also been reported to indicate worse prognosis.^21–23^ In contrast, Crivellaro et al^15^ reported that SUVmax of primary tumor predicted nodal metastases but not recurrence in early-stage uterine cervical cancer.

In addition to the primary tumor, lymph node metastasis is known to be the most important prognostic factor in cervical cancer.^24^ The rate of lymph node metastasis is higher in locally advanced cervical cancer than in early-stage cervical cancer. Accordingly, metabolic activity of metastatic lymph nodes might be important for predicting outcome. Recently, the prognostic significance of the metabolic activity of metastatic lymph nodes was reported in several studies.^25–27^ Onal et al^25^ evaluated the prognostic significance of SUVmax for pelvic lymph nodes in patients with cervical cancer treated with definitive chemoradiotherapy. In their study, pelvic lymph node SUVmax ≥7.5 was found to be a significant prognostic factor for both overall survival and disease-free survival. Akkas et al^27^ also reported that pretreatment pelvic lymph node SUVmax was significantly higher in patients with persistent disease than in patients with no evidence of disease following chemoradiation for FIGO stage IIB-IVB cervical cancer. Unfortunately, all previous studies evaluating lymph nodes focused only on SUVmax.

The limitations of previous studies led us to perform comprehensive analysis of SUVmax, MTV, and TLG of pelvic and/or para-aortic lymph nodes, not just SUVmax. Furthermore, we combined those parameters with those of the primary tumor, creating the summed parameters (SUVmax-S, MTV-S, and TLG-S). Our study revealed that advanced FIGO stage, presence of lymph node metastasis, MTV-T > 47.81 cm^3^, TLG-T > 215.02, MTV-S > 59.01 cm^3^, and TLG-S > 224.15 were all negative prognostic factors for RFS in univariate analysis. After inclusion in multivariate analysis, FIGO stage and MTV-S > 59.01 cm^3^ remained independent prognostic factors of RFS. The advantage of MTV over SUVmax as a predictive tool has been demonstrated for some types of cancer.^21,22,28^ In our study, MTV-S, as a parameter representing the total number of active metabolic tumor cells, showed moderate–to-strong correlation with MTV-T, SUVmax-T, and SUVmax-S. This novel finding indicates the importance of MTV as a predictive parameter and also implies that MTV-S is a useful prognostic indicator predicting recurrence.

We found that SUVmax-T was not a prognostic indicator, which is different from previous findings. Although SUVmax-T is a popular semiquantitative measure, it may not reflect the heterogeneous nature of the primary tumor, especially in large-sized tumors as in our series. In addition, it can be easily affected by statistical noise and pixel size. Unlike our study, previous studies that revealed the association between SUVmax of the primary tumor and poor clinical outcome included patients with early-stage cervical cancer.^17,21,23,^^30^ The discrepancy of our result from the previous studies might be attributed to the enrollment of inoperable patients and exclusion of patients with early stage disease. Furthermore, evidence supporting our results is available in the literature. Akkas et al^27^ reported that tumor SUVmax was not an independent prognostic factor in the prediction of disease recurrence or persistence in patients with inoperable cervical cancer. Another study by Yoo et al^10^ also showed that SUVmax of the primary tumor was not associated with RFS. On the contrary, they suggested that TLG better predicted prognosis than did other PET parameters and FIGO stage was not an independent prognostic factor unlike our results. In this light, there are still controversial results regarding the prognostic value of each PET parameter in predicting disease outcome. Therefore, separate prospective trials with larger series of patients with early-stage cervical cancer and those with advanced-stage cervical cancer are warranted to demonstrate the prognostic value of various PET parameters.

In this study, no PET parameter showed a significant association with OS. However, this result should be interpreted with caution because the optimal cutoff values of PET parameters by ROC curve analysis were initially determined for predicting recurrence, not survival.

Our study has some limitations. First, this is a retrospective study with a limited number of patients from a single center. Second, the median follow-up duration was relatively short. Third, we did not perform histopathological verification of metastatic lymph nodes because PET/CT has been demonstrated to be a reliable method to detect lymph node metastasis.^29^ Nevertheless, our results are noteworthy because this is the first study to elucidate the clinical usefulness of MTV-S for the prediction of RFS in patients with locally advanced cervical cancer who underwent definitive chemoradiotherapy. Prospective analysis with a longer follow-up period and a larger number of patients is warranted.

Our preliminary results reveal that MTV-S is an independent prognostic factor for RFS in patients with locally advanced cervical cancer who are treated by definitive chemoradiotherapy. This may help clinicians administer more intensive treatment and perform close monitoring of patients with high MTV-S.
